# Ultrasound-assisted enzymatic hydrolysis of loach (*Misgurnus anguillicaudatus*) protein for antioxidant peptides: process optimization, structural characterization, and Keap1/Nrf2-mediated antioxidant mechanism

**DOI:** 10.1016/j.ultsonch.2025.107665

**Published:** 2025-11-02

**Authors:** Zhongxing Chu, Guangfan Qu, Feiyan Yang, Zuomin Hu, Feijun Luo, Qinlu Lin

**Affiliations:** a Hunan Provincial Key Laboratory of Deeply Processing and Quality Control of Cereals and Oils, Hunan Provincial Key Laboratory of Forestry Edible Resources Safety and Processing, National Research Center of Rice Deep Process and Byproducts, Central South University of Forestry and Technology, Changsha 410004 Hunan, China; b School of Liquor and Food Engineering, Guizhou Rosa Roxburghii Research Institute/Guizhou Techonicial Innovation Center of Industry, Guizhou University, Guiyang 550025 Guizhou, China

**Keywords:** Loach peptide, Ultrasound-assisted enzymatic hydrolysis, Structural characterization, Antioxidant

## Abstract

Loaches are kinds of freshwater fish rich in protein, which have high development value. Loach protein hydrolysates were prepared by ultrasound-assisted enzymatic hydrolysis. The results showed, the hydrolysate exhibited strong antioxidant activity under the following conditions: ultrasonic power of 200 W, treatment time of 22 min, and neutral enzyme dosage of 10 KU/g. The structure characterization and analysis of hydrolysates under different treatments showed that ultrasound-assisted enzymatic hydrolysis could form more bioactive peptides with hydrophobic amino acids. A total of 2289 peptide sequences were identified, and 8 ideal peptide sequences were screened by combining biological activity score, hydrophobicity and other bioinformatics indicators. The binding energy between loach peptide and Keap1-Nrf2 protein was compared via molecular docking simulation. Among them, the binding energy between FG-14 and Keap1-Nrf2 was the highest (−86.43 kJ/mol). Subsequent experiments further verified the good antioxidant effect of FG-14. This study provides theoretical and technical support for high-value utilization of loaches.

## Graphical abstract

**Figure:**
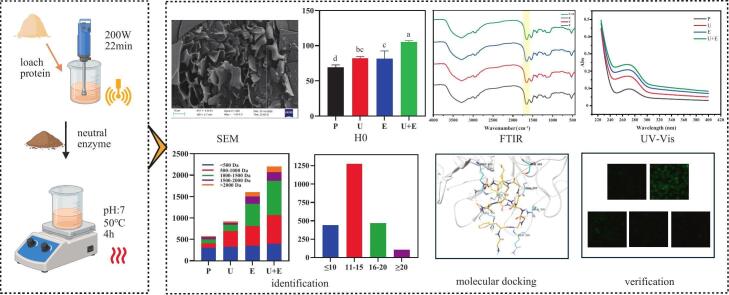

## 1 Introduction

Loach (Misgurnus anguillicaudatus), a common freshwater fish, is widely distributed in China and other parts of East Asia. Loach is rich in high-quality proteins, polysaccharides and vitamins, which have high nutritional value [1,2]. Studies have found that loach peptide has good antioxidant, anti-osteoporosis and anti-fatigue effects [3]. Notably, the antioxidant property of loach peptide has become a prominent research focus in recent years, owing to its capability to effectively modulate oxidative stress and prevent related complications [4]. Antioxidant peptides can reduce free radicals and reactive oxygen species produced by body metabolism, thereby reducing the damage to biological macromolecules such as DNA and proteins [5]. There are many ways to prepare peptides. Enzymatic hydrolysis is characterized by its cost-effectiveness and operational simplicity [6]. Nevertheless, suboptimal enzyme activity and inappropriate operation during the hydrolysis process may induce protein aggregation, which can compromise the efficiency and biological activity of peptide preparation.

Ultrasound, as a typical environmentally friendly extraction technique, is characterized by high efficiency, environmental friendliness, and operational simplicity [7]. The cavitation and mechanical effects induced by ultrasonic vibration are capable of enhancing the contact and interaction between substrate and enzyme, or altering the substrate conformation to further improve the hydrolysis efficiency [8]. Ultrasound-assisted extraction of antioxidant peptides from fermented soybean residue significantly improved the stability and activity of antioxidant peptides [9]. Wei et al. [10] reported that ultrasound-assisted enzymatic hydrolysis of flaxseed protein generated a higher yield of free amino acids, which significantly enhanced the antioxidant capacity of the enzymatic hydrolysate. Ultrasound-assisted enzymatic hydrolysis can not only improve the antioxidant effect of plant protease hydrolysates, but also improve the antioxidant activity of other proteolytic lysates. Ultrasound-assisted hydrolyzed products of goat milk casein showed high solubility and good antioxidant activity *in vitro* [11]. These results suggest that ultrasound may potentiate the antioxidant activity of proteins by promoting reactant contact and inducing conformational changes in protein structures.

Loach has great potential in functional food development due to its high protein content. Existing studies have confirmed the good biological effects of loach peptide [4]. However, the low efficiency of enzymatic hydrolysis and the limited yield of active peptide in traditional enzymatic hydrolysis methods restrict its industrial application. In particular, although the muscle tissue of mud carp is rich in protein and can be initially broken down by proteases, the natural fish protein has a compact spatial structure (such as the α-helix and β-folding assembly of myofibrillar protein), and the connective tissue in the muscle tissue (such as collagen) will encapsulate the protein molecules, making it difficult for proteases to access the internal enzymatic cleavage sites and limiting the hydrolysis efficiency and the yield of active peptides [12]. Ultrasonic-assisted enzymatic hydrolysis technology can destroy the spatial structure of proteins and increase the enzymatic hydrolysis sites by virtue of cavitation effect and mechanical effect, which provides a new idea for the efficient preparation of highly active antioxidant peptides [13]. However, there is a lack of research on ultrasound-assisted enzymatic hydrolysis of loach protein, and its effects on peptide composition, structural characteristics and antioxidant mechanism are not clear. Therefore, in this study, loach protein was extracted via alkali-soluble acid precipitation method, and the preparation conditions of its antioxidant peptides were optimized using single-factor tests and response surface methodology. Thereafter, the loach antioxidant peptides were structurally characterized and their antioxidant activities were analyzed. The findings provide a theoretical foundation for the value-added utilization of loach resources and the development of functional foods.

## 2 Materials and methods

### 2.1 Chemicals and reagents

Freshly deceased loaches were purchased at a local market in Changsha, Hunan Province. Neutral protease (NP), alkaline protease (AP) and papain required for the experiments were purchased from Nanning Doing-higher Bio-Tech Co.,LTD. DPPH and ABTS were purchased from reagents purchased from Beijing Solebo Biotechnology Co., LTD. Loach peptide FG-14 was provided by Shanghai Gil Co., LTD. Fetal bovine serum (FBS) was purchased from Umedium (Hefei, China), and RPMI 1640 was purchased from Gibco-BRL (Carlsbad, CA, USA). All other reagents were analytically pure.

### 2.2 Extraction of loach proteins and preparation of loach peptides

After removing the internal organs and body surface blood water, the loach was ground in a meat grinder, dried at low temperature, and degreased with petroleum ether. The specific operation was as follows: minced meat was mixed with petroleum ether at a ratio of 1:5 (w/v), placed in a constant temperature water bath shaker at 50 °C, and shaken at 150 r/min for 4 h to degrease. After the end of the oscillation, the upper petroleum ether phase was discarded by centrifugation at 8000 × g for 15 min at 4 °C. The above defatting procedure was repeated twice until there was no obvious oil layer in the lower minced meat. The defatted loach powder was obtained by freeze-drying and stored in a desiccator for further use [14]. The crude protein of loach was extracted by alkali dissolution and acid precipitation. Specifically, dried defatted loach powders were mixed with ultrapure water at a 1:10 (w/v) ratio. Referring to previous studies, the crude protein of loach was extracted by alkali-soluble acid precipitation [15]. The pH of the mixture was adjusted to 11.0, and the mixture was magnetically stirred for 60 min at room temperature. Following centrifugation at 10,000 × g for 10 min, the supernatant was collected and its pH was adjusted to 4.5. The solution was allowed to stand undisturbed for 60 min at room temperature, and the precipitate was collected by centrifugation under the same conditions. Crude loach protein was obtained by adding a certain proportion of ultrapure water and adjusting the pH to 7.0, and then dialysis and freeze-drying the solution. According to literature reports, the protein content of freeze-dried loach crude protein samples was determined by Kjeldahl nitrogen determination method, and the results showed that the protein content was 89.23 %±1.56 %, which met the requirements of substrate purity for subsequent enzymatic hydrolysis experiments [16]. According to previous literature [17], crude protein and ultrapure water were mixed at a ratio of 1:20, and 10,000 U (units)/g papain, AP and neutral proteinase were added respectively. The optimal temperature and pH of each enzyme were adjusted. According to literature review, the optimum temperature and pH of papain were 55 °C and 6.0, respectively. The optimum temperature and pH of AP were 50 °C and 9.0, respectively. The optimum temperature and pH of NP were 50 °C and 7.0, respectively [18]. In addition, considering the sensitivity of each enzyme activity, a low concentration of 0.01 mol/L hydrochloric acid and sodium hydroxide solution was selected to adjust the pH of the system [19]. In a temperature-adjustable water bath with constant temperature, using a pH indicator, adjust to the optimal pH for each enzyme. After 4 h, the enzyme was inactivated in a water bath at 100 °C for 10 min. The supernatant was collected by centrifugation.

### 2.3 Determination of protein hydrolysis in loach

According to the existing literature, the degree of hydrolysis of loach protein was evaluated by the o-phthalaldehyde (OPA) method [20]. In brief, the enzymatic supernatant sample solution was mixed with OPA reagent at a ratio of 1:20. The absorbance of the mixture was then recorded at 340 nm.

### 2.4 Determination of the antioxidant capacity of loach peptides in vitro

Referring to previous studies, ABTS, DPPH, and hydroxyl radical scavenging abilities were measured in the protein solution before enzymatic hydrolysis and in the supernatants of the three enzymatic solutions [11].

### 2.5 Single factor experiment

Loach protein of equal weight was weighed and a certain volume of ultrapure water was added to prepare the suspension, and a certain amount of NP was added for enzymatic hydrolysis at pH 7.0 and 50 °C. The temperature was controlled using a constant temperature water bath. The enzyme digestion time was 4 h. According to the following arrangement, different degrees of sonication were performed first, and then the enzymatic hydrolysis reaction was carried out.

The enzyme addition amount was maintained at 10 KU/g, and the ultrasonic power was set at 200 W. The influence of varying sonication durations (0, 10, 20, 30, and 40 min) on the ABTS radical scavenging activity of loach peptides was investigated. (2) Ultrasonic power 200 W and ultrasonic time under the condition of 20 min, studied different enzyme dosage (6, 8, 10, 12, and 14 KU/g) for loach peptide to remove the effects of free radicals ABTS. (3) Under the conditions of enzyme dosage of 10 KU/g, reaction time of 20 min, and ultrasonic time being the same, the effects of different ultrasonic powers (160, 180, 200, 220, and 240 W) on the ABTS free radical scavenging ability of loach peptide was investigated.

The ultrasonic treatment was carried out using a cell disruption instrument (LC-CB-650, Shaoxing, Zhejiang). The volume of each group of loach protein mixture was 20 mL. Since heat generation during ultrasonication may affect protein structure, temperature was controlled via an ice bath combined with real-time thermocouple monitoring, maintaining the system temperature stably at 25 ± 1 °C (room temperature range) to avoid premature changes in protein conformation caused by high temperatures.

### 2.6 Response surface testing and optimization design

In accordance with the aforementioned experimental results, Response surface methodology was employed using Design-Expert 13.0 to optimize three key parameters: sonication time, ultrasonic power, and enzyme loading (Table S1). The ABTS radical cation scavenging activity was adopted as the evaluation criterion to ascertain the optimal process parameters for preparing loach antioxidant peptides via ultrasound-assisted enzymatic hydrolysis.

### 2.7 Particle size and zeta potential

A sample of 1 mg/mL of the liquid to be tested was prepared before measurement. The particle size and Zeta potential of loach protein, loach protein treated by sonication, loach protein treated by enzymatic hydrolysis, and loach peptide treated by both sonication and enzymatic hydrolysis were measured on a Nano-ZS particle size meter and zeta potential analyzer (Nano-ZS 90, Malvern Instruments, UK) at room temperature. The refractive indices of the aqueous phase was 1.330.

### 2.8 Scanning electron microscopy

Scanning electron microscopy (SEM) was employed to characterize the surface morphology of the samples. Powder samples prepared under distinct treatment conditions were mounted onto a sample stage. Following gold sputtering coating, the morphological features of the samples were observed under the SEM (ZEI88 Sigma 360, Germany).

### 2.9 Fourier transform infrared (FTIR) spectroscopy measurement

FTIR spectroscopy was performed by mixing prespecified amounts of samples subjected to different treatments with KBr at a 1:100 (w/w) ratio. The mixtures were then analyzed using an FTIR spectrometer (Vertex 70, Bruker Co., Germany) within a wavenumber range of 500–4000 cm^−1^.

### 2.10 Ultraviolet–visible (UV–Vis) spectroscopy and intrinsic fluorescence measurement

Using ultrapure water as a reference, the UV spectrum was scanned over a wavelength range of 200–400 nm with a UV–Vis spectrophotometer (Agilent, Cary 60, Malaysia). Predefined amounts of samples from different treatment groups were dissolved to prepare 1 mg/mL (w/v) solutions, and their intrinsic fluorescence emission spectra were recorded using a fluorescence spectrophotometer (F-4500, Hitachi, Japan). The excitation wavelength was set at 280 nm, the emission wavelength scanning range was 300–500 nm, the width of excitation slit and emission slit were both 5 nm, and the scanning speed was 100 nm/min.

### 2.11 Determination of surface hydrophobicity

Referring to the existing literature [21], 0.0081 g ANS (1-anilinyl naphthalene-8-sulfonic acid) was weighed accurately, dissolved in 0.01 mol/L phosphate buffer (pH 7.0) and constant volume to 100 mL to prepare a concentration of 200 μmol/L ANS stock solution, which was stored in a dark place at 4 °C and returned to room temperature before use. The sample diluents with different concentrations (0.1–1 mg/mL) were mixed with ANS stock solution at a ratio of 1:1 (v/v), left in the dark for 15 min, and the fluorescence intensity was measured by a fluorescence spectrophotometer. The fluorescence intensity of the resulting mixture was evaluated at an excitation wavelength of 390 nm and an emission wavelength ranging from 400 to 600 nm. After plotting the fluorescence intensity against the corresponding protein concentration, the slope of the linear fit was defined as an indicator of surface hydrophobicity.

### 2.12 Determination of free sulfhydryl content

The sulfhydryl content was determined according to the method reported in the literature [22]. In brief, the fully dissolved protein solution was mixed with 5,5′-Dithiobis (2-nitrobenzoic acid) (DTNB) solution, followed by incubation at room temperature in the dark for 1 h. The absorbance at 412 nm was measured using a UV–Vis spectrophotometer to calculate the sulfhydryl group content.

### 2.13 X-ray diffraction (XRD) measurement

The XRD patterns of loach proteins and the protein treated in different ways were determined by X-ray diffractometer (EQUINOX 100, Thermo Fisher, America). Patterns were acquired at a scan rate of 4°/min in the 2θ region from 5° to 90°.

### 2.14 Differential scanning calorimetry (DSC) measurement

The lyophilized powder (20 mg) from different treatments of loach protein was placed in an aluminum crucible under a continuous flow of oxygen. The thermal stability of samples was evaluated by DSC (DSC 214 Polyma, NETZSCH, Germany).

### 2.15 Identification and bioinformatics analysis of loach peptides

Referring to previous studies [23], desalting was first performed using a desalting column. Subsequently, LC-MS/MS mass spectrometry analysis system (RIGOL L-3000, Beijing, China) was used for peptide isolation and identification. MaxQuant 2.4.14.0 was used for data analysis. Bioinformatics analysis is now widely used for the screening and application of active peptides [24]. Using PeptideRanker (https://distilldeep.ucd.ie/PeptideRanker/) predict the biologic activity of the active peptide. The hydrophobicity of potentially bioactive peptides was predicted by (https://pepcalc.com/). Use online software ToxinPred analysis peptide sequence toxicity (https://webs.iiitd.edu.in/raghava/toxinpred/index.html). AllerTOP online software was used to predict the allergenicity of peptides (https://www.ddg-pharmfac.net/AllerTOP). AnOxPePred to evaluate radical scavenging activity (https://services.bioinformatics.dtu.dk/service.php). Peptides with good biological activity, high hydrophobicity, non-toxicity, harmlessness and good antioxidant effect were selected for the next test.

### 2.16 Molecular docking

AlphaFold3 was used to investigate the mechanism of action and interaction between the ligand and the receptor. Natural protein structure of PBD files can be from EBI (https://alphafold.ebi.ac.uk), protein information can also be through UniProt (https://www.uniprot.org). Prediction of access AlphaFold3 (https://deepmind.google/technologies/alphafold/). PyMOL (https://pymol.org/2/) can visualize the predicted structures.

### 2.17 Cell experiment

#### 2.17.1 Cell viability assay

To evaluate the good antioxidant effect of the peptide sequences screened above, we used the HUVEC model of oxidative damage by H_2_O_2_. Sample safety needs to be assessed before formal experiments. Referring to previous studies [25,26], cells were treated with different concentrations of peptides (0–200 μg/mL). The cell morphology was observed and photographed under an inverted fluorescence microscope (DMI3000 B, Lecia Microsystems CMS GmbH, Germany).

#### 2.17.2 Determination of intracellular ROS

After the loach peptide was confirmed to be non-toxic to cells, the intracellular ROS level was evaluated using the ROS detection kit. Referring to previous studies [27], after HUVEC were treated with H_2_O_2_ and loach peptide at different concentrations for 16–18 h, ROS levels in different groups were detected according to the instructions of the kit, and cell morphology was observed under an inverted fluorescence microscope and photographed (Ti2-E, Nikon, Japan).

#### 2.17.3 Studies on expression of oxidation-related proteins and genes

Referring to previous study [27], western blot and RT-qPCR were used to investigate the expression of oxidation-related proteins and genes in cells treated with H_2_O_2_ and loach peptide.

### 2.18 Data processing and analysis

Statistical analysis was performed using SPSS 22.0 software, and results were replicated at least three times and expressed as mean ± SD. Analyses included one-way ANOVA followed by Duncan's multiple range test. Significant differences were considered to be *p* < 0.05.

## 3 Results and discussion

### 3.1 Screening of proteases

**Fig. 1:**
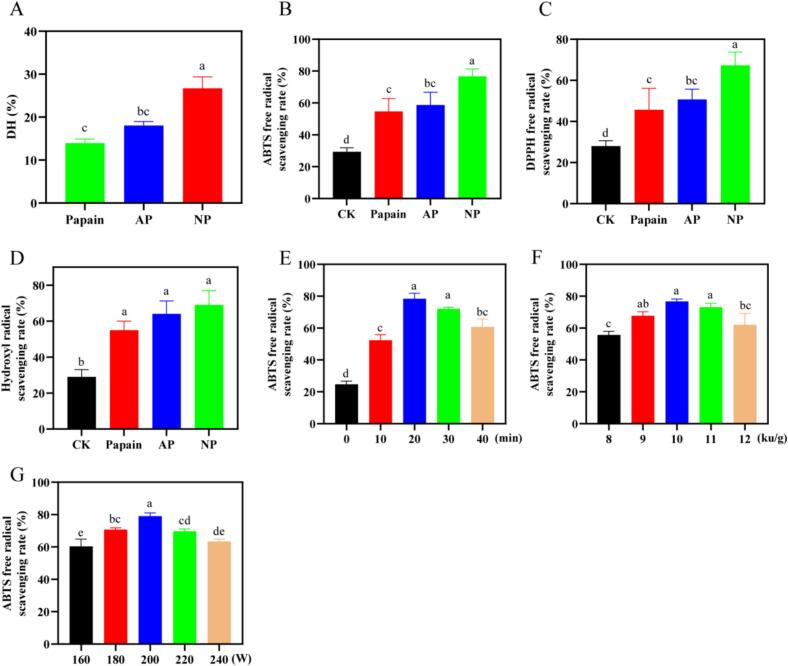
(A) Effects of different enzymes on proteolysis of loach; (B-D) Effect of different enzymatic treatments on ABTS, DPPH and hydroxyl radical scavenging; (E-G) Effects of different sonication times, enzyme doses, and ultrasound power on radical scavenging in ABTS. (AP:Alkaline protease; NP: Neutral protease).

In the process of proteolysis, different proteases show significant differences in efficiency and antioxidant activity of derived peptides. Based on published literature and preliminary experimental data, AP, NP, and papain were selected for the hydrolysis of loach proteins [3,28]. As indicated by the analysis of degree of hydrolysis and three radical scavenging activities in Fig. 1, the hydrolysate obtained by NP exhibited the highest hydrolysis degree and the strongest antioxidant capacity. The degree of hydrolysis for neutral enzymatic hydrolysis was 47.93 % and 91.58 % higher than that of AP and papain, respectively. The ABTS free radical scavenging ability of the hydrolysates of NP was 76.67 ± 4.73 %, the DPPH free radical scavenging rate was 67.33 ± 6.43 %, and the hydroxyl free radical scavenging rate was 69.00 ± 7.94 %, which demonstrated the good antioxidant effect of NP. As an endonuclease, NP has the characteristic of low specificity and can cut a large number of terminal amino acid residues, thus making the hydrolysate have a strong ability to scavenge free radicals. This is consistent with the research of Huo and colleagues [29], who pointed out that the hydrolysis of walnuts hydrolysate by NP has good hydrolysis degree and free radical scavenging activity. NP was selected for the further study.

### 3.2 Analysis of single factor experimental results

ABTS radical scavenging was determined by sonication at a fixed enzyme dose of 10 KU/g and an ultrasound power of 200 W for different periods of time. According to the results of section 3.1, the ABTS radical scavenging activity of the NP hydrolysate was the highest. Therefore, the ABTS scavenging rate was chosen as the primary optimization index. According to the results in Fig. 1E, within the first 20 min, with the increase of different ultrasonic time, the ABTS free radical clearance rate of the loach protease solution increased continuously, and the clearance rate increased by 2.18 times compared with the group without ultrasonic treatment. However, after 20 min of sonication, with the increase of sonication time, the free radical scavenging rate showed a downward trend, and the free radical scavenging rate showed a significant difference between 20 min and 40 min of treatment. The above results indicated that excessive ultrasonic treatment will lead to a decrease in ABTS free radical scavenging capacity of loach protein hydrolysate, and the antioxidant effect will be inhibited. Therefore, 20 min of sonication was determined to be the optimal time for this process.

The ultrasound power was set at 200 W and the sonication time was 20 min. ABTS radical scavenging ability was analyzed at different enzyme doses. As shown in Fig. 1F, the antioxidant capacity of loach protease hydrolysates reached 76.67 ± 1.53 % when the amount of enzyme was 10 KU/g, indicating that appropriate amount of protease could hydrolyze loach proteins into peptides, thereby enhancing its antioxidant activity. However, studies have found that when the amount of enzyme added exceeds a certain range, the protein substrate is fully hydrolyzed, which will lead to the further degradation of the enzymatic hydrolyzed peptide into lower structure amino acids, which will also reduce the antioxidant activity of the system [12]. This is consistent with the study of Islam et al. [30], which showed that the antioxidant effect of soybean protease hydrolysates decreased significantly as the amount of soybean protease added increased. This is related to the continuous increase of free amino acids in the system. Therefore, an enzyme dose of 10 KU/g was determined to be optimal.

The enzyme dose was optimized to 10 KU/g and sonicated for 20 min at different powers. As shown in Fig. 1G, with the increase of ultrasound power, the free radical scavenging ability of enzymatic hydrolytic ABTS reached a peak of 79.00 ± 2.00 % after treatment with 200 W ultrasound power. Appropriate intensity of ultrasound power can promote bubble formation, and the strong micro-shear jet generated when the bubble implosions can enhance polymer degradation and solvent penetration. On the contrary, if the ultrasonic energy is too strong, it will cause the cavitation bubble to spread rapidly, and then hinder the effective conduction of the ultrasonic energy to the medium. After appropriate intensity ultrasonic treatment, the extraction rate of polysaccharides from coix seeds increased significantly. However, after excessive ultrasonic treatment, the extraction rate decreased to different degrees, which may be due to the cavitation of bubbles will hinder the conduction of energy and affect the efficiency of polysaccharide extraction [31]. Notably, moderate ultrasonic power treatment can facilitate unfolding of protein structure, thereby exposing more enzymatic cleavage sites and hydrophobic regions, which contribute to the generation of peptides with potent antioxidant activity. It is critical to emphasize that excessively high ultrasonic power generates substantial heat and energy, which may disrupt protein tertiary structures, inducing denaturation and aggregation. Such structural alterations impede direct enzyme-substrate interaction and compromise the efficiency of enzymatic hydrolysis. This result is consistent with the study by Cao et al., [32] which showed that the antioxidant effect of quinoa protein hydrolysate was inhibited with increasing ultrasonic power. Given these findings, 200 W was determined to be the optimal ultrasound power.

### 3.3 RSM model design and result analysis

Based on the findings from the single-factor experiments, the preliminary optimal conditions, the preliminary optimal conditions for ultrasound-assisted enzymatic hydrolysis of loach proteins were determined as: sonication time of 20 min, NP dosage of 10 KU/g, and ultrasonic power of 200 W. To further optimize these parameters, multivariate analysis was conducted using Design-Expert 13 software (Table S2), yielding a binary polynomial regression equation for the ABTS radical scavenging activity (P) as a function of sonication time (A), ultrasonic power (B), and enzyme loading (C):

$${P=77.02+1.95\times A+1.20\times B-0.15\times C+1.21\times AB+0.28\times AC-0.34\times BC-5.98\times A}^{2}-4.30\times B^{2}-3.08\times C^{2}$$

**Fig. 2:**
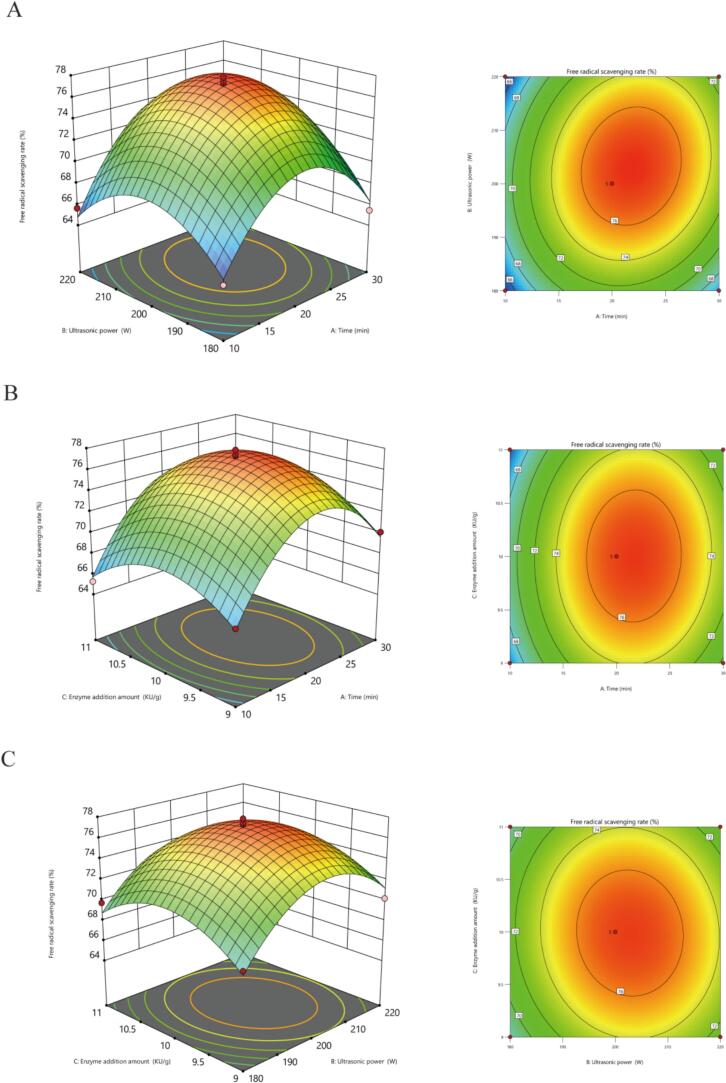
Effect of interaction of different factors on free radical scavenging rate of loach protein hydrolysate ABTS.

As shown in Table S3, the regression model exhibited a coefficient of determination R^2^ = 0.9807, indicating an excellent fit between measured and predicted ABTS scavenging values. The adjusted R^2^ = 0.9558 suggested that the model accounted for 95.55 % of the response variable variance. The model's *p* < 0.01 confirmed its statistical significance. The high goodness-of-fit of the model validated its suitability for modeling and predicting the influences of sonication time, ultrasonic power, and enzyme concentration on ABTS radical scavenging activity. Fig. 2 illustrated that the interactive effects of sonication time-ultrasonic power, sonication time-enzyme loading, and ultrasonic power-enzyme loading significantly influenced ABTS scavenging efficiency. Analysis using Design-Expert 13 software predicted the optimal process parameters as follows: sonication time of 21.80 min, ultrasonic power of 203.29 W, and enzyme loading of 9.99 KU/g, with a predicted ABTS radical scavenging rate of 77.29 %. Adjusting for practical feasibility, the optimized conditions were set as 22 min sonication, 200 W ultrasonic power, and 10 KU/g enzyme dosage. Under these conditions, the experimental ABTS scavenging rate reached 77.04 ± 1.34 %, showing no significant difference from the predicted value. This consistency validated the regression equation's reliability and the feasibility of RSM-optimized conditions.

### 3.4 The influence of ultrasound-assisted enzymatic hydrolysis on the particle size and zeta potential of loach protein

**Fig. 3:**
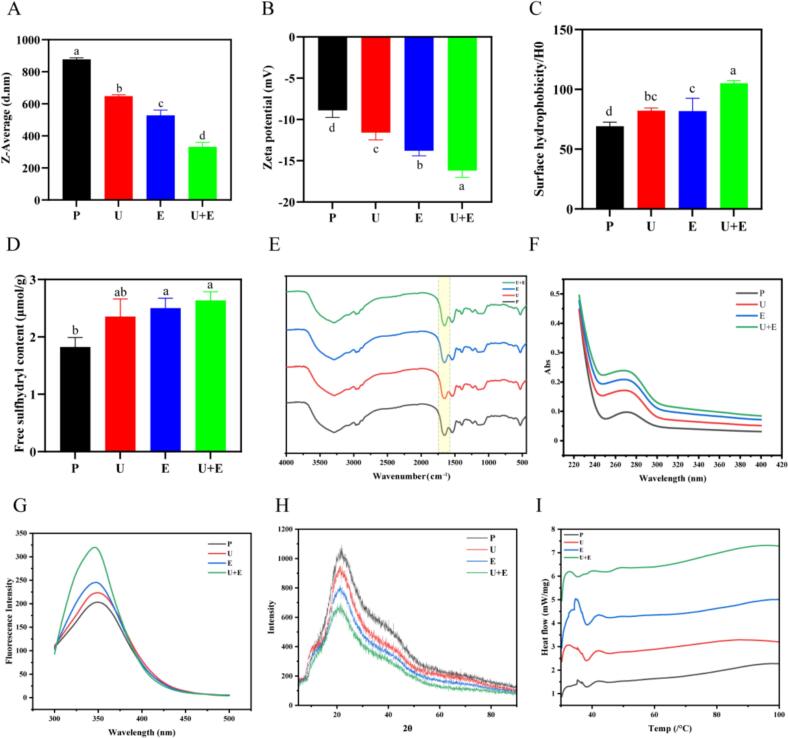
(A-D) The particle size, Zeta potential, surface hydrophobicity and free sulfhydryl content of loach protein and its enzymatic hydrolysates under different treatment conditions; (E-G) Infrared spectrum, ultraviolet spectrum and fluorescence spectrum of loach protein and its enzymatic products under different treatment conditions; (H) X-ray diffraction pattern; (I) Differential calorimetric scan map. (P: protein; U: Ultrasound treatment; E: Enzymatic hydrolysis; U + E: Ultrasound-assisted enzymatic hydrolysis).

As shown in Fig. 3A, compared with loach protein, the average particle size of protein decreased significantly from 876.90 ± 10.41 nm to 331.50 ± 27.20 nm after ultrasonic treatment, enzymatic hydrolysis treatment and simultaneous treatment with ultrasound-assisted enzymatic hydrolysis. This finding was similar to that of Peng [33]. Sonication can improve the efficiency of the enzymatic hydrolysis process. Sonication effectively disrupts structural interactions such as intermolecular hydrogen bonds, electrostatic and hydrophobic interactions of proteins, resulting in a reduction in particle size. The reduction of particle size can significantly increase the specific surface area of the peptide, enhance the contact efficiency with free radicals, and improve the antioxidant effect.

Zeta potential quantifies the electrostatic charge of particles in solution, where a higher absolute zeta potential indicates greater colloidal stability [34]. As shown in Fig. 3B, the native loach protein exhibited a zeta potential of −8.90 ± 0.85 mV. Ultrasonication alone shifted the zeta potential to a more negative value of −11.60 ± 0.87 mV, while enzymatic hydrolysis alone resulted in a further decrease to −13.80 ± 0.61 mV. Notably, ultrasound-assisted enzymatic hydrolysis caused the zeta potential to decrease to −16.20 ± 0.82 mV. These results suggest that ultrasonic and enzymatic treatments induce structural modifications in the protein, leading to conformational loosening, altered surface charge distribution, and enhanced particle stability and dispersibility. Increasing the absolute value of Zeta potential can reduce peptide intermolecular aggregation and improve water solubility, thereby enhancing antioxidant activity.

### 3.5 Effect of ultrasound-assisted enzymatic hydrolysis on the microstructure of loach proteins

**Fig. 4:**
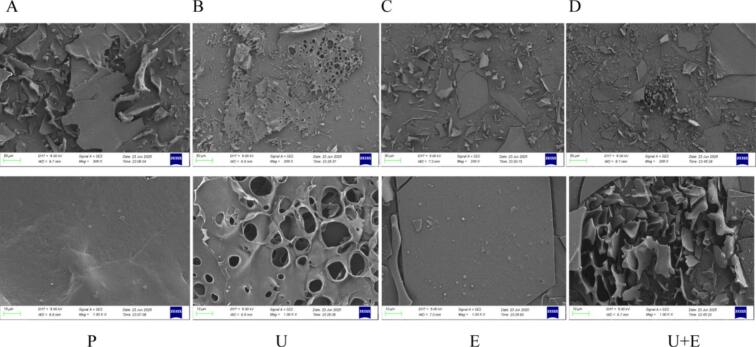
SEM structure diagrams of loach protein and its enzymatic hydrolysates under different treatment methods. (P: protein; U: Ultrasound treatment; E: Enzymatic hydrolysis; U + E: Ultrasound-assisted enzymatic hydrolysis).

SEM was used to observe the microstructure of loach proteins and their enzymatic hydrolysates under different treatment conditions, as shown in Fig. 4. When the magnification was 200 times, the loach protein was observed to be larger in size and more compact in structure. After ultrasonic treatment, a large number of holes appeared in the loach protein particles. The loach protein became smaller after enzymatic hydrolysis. Especially after ultrasound-assisted enzymatic hydrolysis, the loach protein structure was loose and the particles were evenly distributed. Using 1000 x magnification, microscopic changes in the structure of loach protein was further revealed. After sonication, the protein appeared a loose and porous structure, and the particles appeared small and irregular. This change favored the enzyme-substrate interaction, resulting in smaller particles with uniform distribution and a loose porous structure that was enzymatically hydrolyzed after simultaneous sonication and enzyme treatment. The changes in the microstructure of loach protein are consistent with previous findings reported in the literature for cottonseed protein [35] and black bean protein [36]. Under the transient high pressure and shear forces induced by ultrasound, the tertiary structure of the protein is disrupted, thereby exposing more hydrophobic groups and buried internal regions. This leads to an increase in the surface area and active sites available for enzyme-protein interaction. Consequently, sonication facilitates enzymatic hydrolysis and promotes the generation of smaller bioactive peptides.

### 3.6 Effect of ultrasound-assisted enzymatic hydrolysis on surface hydrophobicity and free sulfhydryl groups of loach proteins

Surface hydrophobicity serves as an indicator of hydrophobic group exposure on the surface of protein molecules, which can indirectly reflect changes in protein structure [27]. The hydrophobicity of loach protein under different treatment conditions was shown in Fig. 3C. The H0 of ultrasound-assisted enzymatic hydrolysis was 105.07 ± 3.26, which was significantly higher than that of other groups. The increase in hydrophobicity may be attributed to the destruction of the complex structure of the protein by the cavitation effect triggered by ultrasound. The forces between and within the protein molecules are disrupted, facilitating enzyme and protein interactions, thereby exposing a large number of internal hydrophobic groups. The increased surface hydrophobicity is due to the hydrophobic amino acid residues (e.g. Leu, Phe) exposed by ultrasound, which can bind free radicals through hydrophobic interaction, further supporting the positive correlation between hydrophobicity and antioxidant activity. The above results are consistent with the already reported findings of Duan et al, [37]. The surface hydrophobicity of loach peptide is directly related to their antioxidant activity-hydrophobic amino acid residues (Leu and Phe) can bind free radicals through hydrophobic interaction, and enhanced hydrophobicity can improve the adsorption ability of peptides on cell membranes and promote their entry into cells to exert antioxidant effects.

The group of free sulfhydryl groups plays a crucial role in protein folding and stability, and will affect the structural and interfacial properties of proteins. As shown in Fig. 3D, the free sulfhydryl content of loach protein was significantly increased by 29.12 % after ultrasonic treatment. Compared with the untreated group, the free sulfhydryl content of enzyme hydrolysis alone group and ultrasound-assisted enzymatic hydrolysis group was significantly increased, which may be due to cavitation, mechanical and thermal effects, and enzyme hydrolysis, leading to changes in the internal structure of the protein and the exposure of internal −S-H [38]. However, there was no significant difference in free sulfhydryl group content between ultrasound-assisted enzymatic hydrolysis and ultrasound-assisted enzymatic hydrolysis. This may be because the protein may form disordered aggregates after ultrasonic treatment, and some sulfhydryl groups are re-encapsulated in the aggregates. At this time, the enzyme could solve the difficulty to contact the newly buried sulfhydryl group, resulting in the release of sulfhydryl group, which was not different from that of the ultrasound alone group [39]. In addition, sulfhydryl groups can scour free radicals by providing electrons (such as converting · OH to H_2_O), and the change in sulfhydryl group content can reflect the effect of ultrasonic-enzymatic hydrolysis on the cleavage of protein peptide bonds and structural unfolding [38], which indirectly indicates the production efficiency of active peptides.

### 3.7 Analysis of infrared spectroscopy results

Infrared spectroscopy is often used to analyze protein secondary structure. The amide I region spans from 1600 to 1700 cm^−1^ in conventional protein secondary structures and is important for protein structure analysis [27]. The additional amide I region is usually directly related to the conformation of the polypeptide chain. Fig. 3E showed that the span of 1500 to 2000 cm^−1^ had absorption peaks, which may be C = O stretching vibrations. Different curves had different peak shape and intensity here. For example, the absorption of pure protein curve was significant, indicating that the chemical bond interaction is stronger when it containeed carbonyl group. The peak shape changed in different ways, or the carbonyl environment was changed due to molecular interaction. These results indicated that ultrasound promoted the secondary structure unfolding of loach protein. This finding was corroborated by Huo et al., [29] in their study on sonicated walnut proteins.

### 3.8 Analysis of UV–vis and fluorescence spectra

UV–Vis spectroscopy and intrinsic fluorescence spectroscopy are primarily employed to analyze the variations in phenylalanine, tryptophan (Trp), and tyrosine (Tyr) contents within proteins. As depicted in Fig. 3F, the UV spectrum exhibited an absorption peak at 270 nm. In accordance with existing literature, the emergence of this peak is primarily attributed to the combined absorption effect of tyrosine and phenylalanine [40]. The UV absorption intensity of loach protein treated with ultrasound assisted enzymatic hydrolysis was higher than that of untreated group or even the group treated with ultrasound or enzymatic hydrolysis alone, indicating that ultrasound and enzymatic hydrolysis destroyed the joint interaction structure between protein molecules, leading to the exposure of more easily degradable internal groups and binding sites, and increasing the content of luminescent groups in the protein. Cui et al., [41] have shown that enzymatic hydrolysis of milk proteins disrupts the internal structure, exposing more hydrophobic and luminescent groups, and that the relative fluorescence intensity depends on the specificity of each enzyme. Noman and colleagues found that the internal intermolecular interactions within the proteins of Chinese sturgeon (*Acipenser sinensis*) were disrupted by sonication [17]. Under the action of cavitation and mechanical forces generated by ultrasound, more hydrophobic groups are exposed, thus increasing the intensity of UV absorption.

Intrinsic fluorescence intensity serves as a proxy for the exposure degree of tryptophan and tyrosine residues, capable of reflecting intramolecular interactions and conformational transitions, thereby indicating alterations in protein tertiary structure [42]. Fig. 3G presents the intrinsic fluorescence spectra of loach protein under different treatment groups. Consistent with the UV–Vis spectral results, both ultrasonic and enzymatic treatments enhanced the intrinsic fluorescence intensity of loach protein. This enhancement is attributed to the exposure of buried fluorescence-sensitive groups during enzymatic hydrolysis, a process further potentiated by the synergistic effect of ultrasonic cavitation [25,26].

### 3.9 The analysis of XRD

The effects of ultrasonic and enzymatic treatments on the crystal structure of loach protein were investigated by XRD. Fig. 3H showed the XRD of loach protein under different treatment conditions. The diffraction peak position was 21.80° under different treatment conditions, and the 2θ was generally close, indicating that the characteristic diffraction angle of the basic crystalline phase and the main crystal structure was not greatly changed by the treatment. The diffraction peak intensity of loach protein was the highest, and decreased after ultrasonic and enzymatic treatments, indicating that different treatments have an effect on the crystallicity of loach protein, which may reduce the order of the crystal and lead to a weakening of the diffraction intensity. Additionally, a distinct correlation was observed between peak intensity and particle size. The crystal dimensions influenced the diffraction angle and peak intensity, thereby shedding light on potential conformational alterations in the protein structure and their intermolecular interactions [43]. Ultrasonic and enzymatic treatments reduced the particle size of loach protein (Fig. 3A). Similar to the study of Li et al. [44], as the protein particle size of different treatment groups changed, their XRD peaks also changed. The XRD patterns help to elucidate the structural changes of loach proteins after ultrasound-assisted enzymatic hydrolysis treatment. The reduction of crystallinity indicates that the protein structure changes from order to disorder, which can reflect the damage degree of the spatial structure of the protein caused by ultrasound, and the disorder of the structure helps the protease to contact the enzymatic hydrolysis site, so the XRD results can assist in explaining the mechanism of the improvement of hydrolysis efficiency by ultrasound.

### 3.10 The analysis of thermodynamic properties

DSC is used to study the thermal stability of the complex, and the DSC curve reflects the heat flow of the sample as a function of temperature [45]. Fig. 3I showed that the heat flow value of untreated loach protein was the lowest as a whole, and there was a small thermal disturbance in the low temperature region (20–40 °C), and the heat flow slowly rose with the increase of temperature, indicating the basic characteristics of thermal stability, the intermolecular force and aggregation state of protein molecules were relatively “inherent”, and the thermal transition process was gentle. After ultrasound-assisted enzymatic hydrolysis, there was a strong endothermic peak at low temperature, and the heat flow value was the highest, indicating that the synergistic effect of ultrasound-assisted enzymatic hydrolysis caused deep destruction of protein structure (including aggregation state and molecular conformation), and triggered a large number of endothermic processes (such as more secondary bond breaking and complex aggregation state disintegration) at low temperature. Subsequently, the heat flux continued to rise and remained at a high level, reflecting that the thermal transition process was more complex, and the interaction between protein molecules (peptides) became more susceptible to temperature due to the double treatment. Combined with the previous published literature, the double treatment of ultrasonic and enzymatic hydrolysis enhanced thermal stability of mayonnaise [46,47]. By measuring the change temperature and the change in enthalpy, the thermal stability of proteins/peptides can be reflected. The changes in temperature and enthalpy caused by ultrasound-assisted enzymatic hydrolysis can be used to reflect the molecular mass distribution and structural compactness of peptide segments, and can also provide a reference for the subsequent processing and application of active peptides (such as in food heat processing) [45].

### 3.11 Identification and bioinformatics analysis of loach peptides

**Fig. 5:**
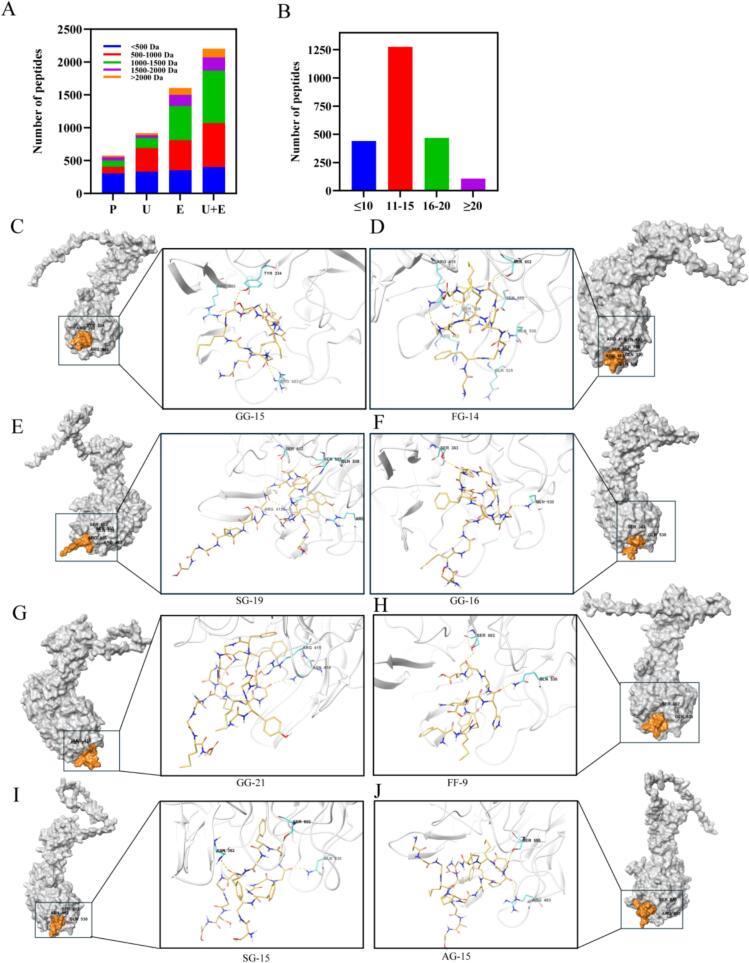
(A) Molecular weight distribution of loach peptides; (B) Statistical chart of amino acid number of loach peptide; (C-J) Docking maps of different loach peptide sequences and Keap1-Nrf2 receptor.

To further explore the antioxidant active components present in the ultrasound-assisted enzymatic hydrolysis of loach proteins, the peptide sequences were qualitatively analyzed using LC-MS/MS. The molecular weight of peptides was closely related to their biological activity. Peptides with low molecular weight had higher antioxidant activity and strong free radical scavenging ability due to their high oligopeptide content [48]. Fig. 5A showed data analysis of the identified peptide sequences. A large number of bioactive peptides were obtained by ultrasonic and enzymatic hydrolysis, especially ultrasound-assisted enzymatic hydrolysis. The loach protein mainly contained < 500 Da active peptide, which may be mainly composed of small molecular amino acids. After ultrasound-assisted enzymatic hydrolysis, the amount of small molecular peptides did not increase significantly, and mainly increased > 500 Da peptide sequences. We speculated that ultrasound-assisted enzymatic hydrolysis could promote the generation of peptides in the range of > 500 Da. This was similar to the study of Huo et al. [29].

As shown in Fig. 5B, the length of these peptides was mainly 11–15 amino acids, accounting for 55.70 %, followed by peptides < 10 and 16–20 amino acids, accounting for 19.22 % and 20.45 %, respectively, and peptides > 20 amino acids accounted for the least. This was mainly related to the restriction sites of neutral enzymes. The high frequency of cleavage by neutral enzymes resulted in difficult retention of long peptides. In addition, the reaction conditions (e.g., pH, temperature) of neutral enzymes may optimize the production efficiency of medium-length peptides. Antioxidant peptides as an important bioactive function, so the biologic activity of the peptides score is of great significance for screening of biological active peptide. A total of 20 peptides with high scores were identified by screening methods including biological activity score, toxicity, hydrophobicity score, and antioxidant activity, as shown in Table S4.

### 3.12 Molecular docking

Keap1-Nrf2 signaling pathway is an important signaling pathway related to oxidative stress injury. Studies have found that bioactive peptides can reduce the ROS level in the body by binding to Keap1-Nrf2 protein and promoting the expression of antioxidant enzymes [49]. Molecular docking is mainly used to predict and analyze molecular interactions. Table S5 and Fig. 5C–J showed the binding energy and molecular docking diagram of loach peptide and Keap1-Nrf2 protein complexes respectively. According to the results, the binding energy of eight loach peptide sequences and Keap1-Nrf2 protein were all negative, indicating that the eight loach peptide sequences could bind to Keap1-Nrf2 targets. Loach peptides bound closely to protein targets, indicating that loach peptides have a strong ability to interact with Keap1-Nrf2 target proteins, which further indicates that these peptides have good antioxidant effects.

### 3.13 The analysis of the antioxidant effect of loach peptide

**Fig. 6:**
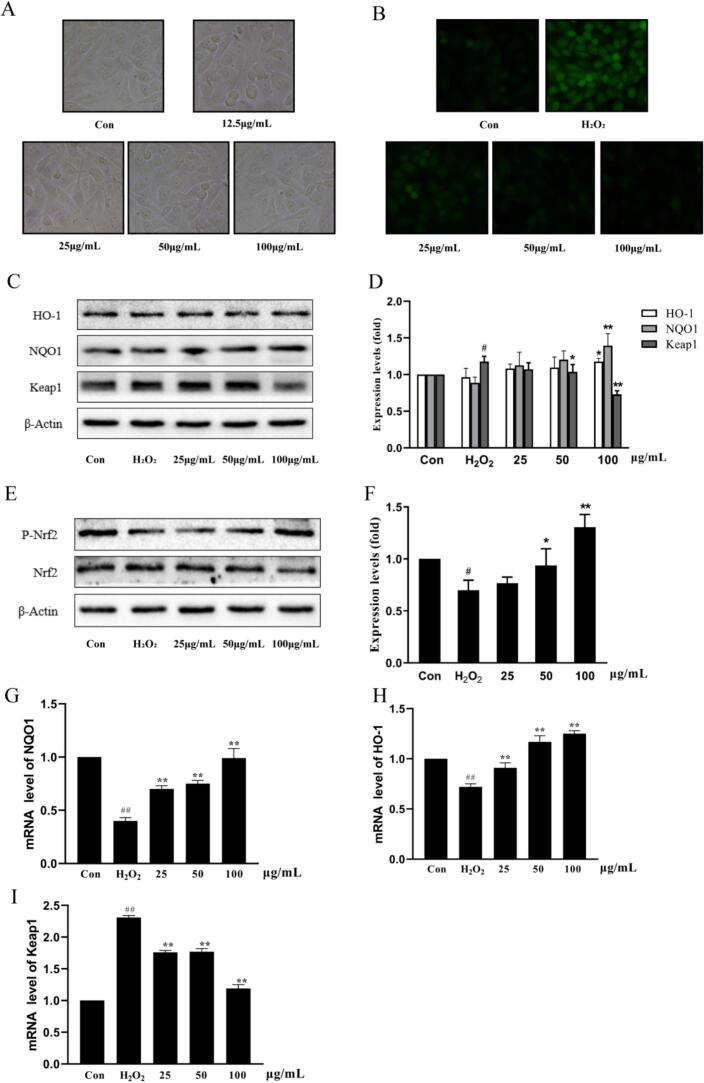
(A) Morphological diagram of HUVEC cells treated with different concentrations of loach peptide; (B) Diagram of ROS in cells treated with different concentrations of loach peptide; (C-D) Protein bands and quantitative analysis of Nrf2 downstream targets HO-1, NQO1, and Keap1; (E-F) Protein bands and quantitative analysis of Nrf2 and p-Nrf2; (G-I) Analysis of mRNA expression of three oxidation-related genes.

According to Table S5, the highest binding energy of FG-14 and Keap1-Nrf2 −86.43 kJ/mol, we speculated that FG-14 had a good antioxidant effect. In Fig. 6A, cell morphology and viability were not affected by FG-14, indicating that FG-14 was a safe and non-toxic bioactive peptide, which verified the predicted results of molecular docking. Fig. 6B showed that FG-14 was able to reduce the ROS content level in the H_2_O_2_-induced cell oxidative damage model in a gradient dependent manner, indicating that FG-14 had a good antioxidant effect, which verified the previous screening and prediction. Similar to the study by Niu et al [21], the novel antioxidant peptide in peanut meal reduced the ROS level in HepG2 cells induced by H_2_O_2_. The results showed that H_2_O_2_ inhibited the downstream protein and gene expression of Nrf2 and increased the expression level of Keap1, but FG-14 could significantly reduce the effect of H_2_O_2_ on protein expression. In addition to remaining silent by binding to Keap1, Nrf2 is also activated by phosphorylation. After the addition of FG-14, the protein expression level of p-Nrf2 increased in a dose-dependent manner (Fig. 6). In summary, FG-14 could increase the protein expression of Nrf2 and its downstream antioxidant genes of heme oxygenase-1 and NADH quinone oxidoreductase 1, and inhibit the expression of Keap1 to exert antioxidant effect, which is consistent with the previous molecular simulation results.

## 4 Conclusions

In this study, the free radical scavenging rate of ABTS was used as the evaluation index of antioxidant effect, and the preparation process conditions of antioxidant peptide of Loach assisted by ultrasound were optimized through the joint analysis of single factor and response surface optimization. The results showed that the loach protease hydrolysates prepared under the optimal conditions using NP, ultrasonic treatment time of 22 min, ultrasonic power of 200 W, and enzyme dosage of 10 KU/g had good antioxidant effect. The structural changes of Loach proteins after different ultrasonic and enzymatic treatments as well as their combined treatments were further analyzed to elucidate the underlying mechanism of the enhanced antioxidant activities of loach protease hydrolysates. Studies have shown that ultrasound-assisted enzymatic hydrolysis can generate more bioactive peptides with C-terminal hydrophobic amino acids, which can enhance antioxidant activity to a certain extent. A total of 2289 peptide sequences were obtained from the ultrasonic treated loach protease hydrolysates. According to the common screening methods of bioinformatics, 20 sequences with high comprehensive scores were selected. Among them, eight sequences including FG-14 showed strong binding to Keap1-Nrf2 protein. The antioxidant effect of loach peptide was further verified by in vitro cell oxidative damage model. The results provide a theoretical basis for the preparation of antioxidant peptides and the development of loach industry.

## CRediT authorship contribution statement

**Zhongxing Chu:** Writing – original draft, Resources, Methodology. **Guangfan Qu:** Software. **Feiyan Yang:** Software, Investigation. **Zuomin Hu:** Validation, Supervision. **Feijun Luo:** Writing – review & editing, Funding acquisition, Conceptualization. **Qinlu Lin:** Writing – review & editing.

## Declaration of competing interest

The authors declare that they have no known competing financial interests or personal relationships that could have appeared to influence the work reported in this paper.
