# Supplementary material for: Ultrasound-assisted enzymatic hydrolysis of loach (Misgurnus anguillicaudatus) protein for antioxidant peptides: process optimization, structural characterization, and Keap1/Nrf2-mediated antioxidant mechanism
Source: Ultrason Sonochem. 2025 Nov 2;123:107665. doi: 10.1016/j.ultsonch.2025.107665 (PMC12639302; doi:10.1016/j.ultsonch.2025.107665)
Supplement: Supplementary Data 1 [file mmc1.docx]

**Supplementary data**

Table S1 Factors and levels of Box-Behnken experiment design

| Factor | Levels | | |
| --- | --- | --- | --- |
|  | -1 | 0 | 1 |
| A: Time (min) | 10 | 20 | 30 |
| B: Ultrasonic power (W) | 180 | 200 | 220 |
| C: Enzyme addition amount (KU/g) | 9 | 10 | 11 |

Table S2 The Box-Behnken experimental design and results

| Number | Time (min) | Ultrasonic power (W) | Enzyme addition amount (KU/g) | DPPH radical scavenging rate (%) |
| --- | --- | --- | --- | --- |
| 1 | 0 | 0 | 0 | 76.05 |
| 2 | 0 | -1 | -1 | 68.52 |
| 3 | 1 | 0 | -1 | 70.12 |
| 4 | 0 | 0 | 0 | 76.53 |
| 5 | 0 | 1 | -1 | 70.21 |
| 6 | -1 | 0 | 1 | 65.27 |
| 7 | 0 | 0 | 0 | 77.28 |
| 8 | -1 | 0 | -1 | 66.45 |
| 9 | 1 | 0 | 1 | 70.03 |
| 10 | 1 | 1 | 0 | 71.63 |
| 11 | 0 | -1 | 1 | 69.77 |
| 12 | -1 | -1 | 0 | 64.29 |
| 13 | -1 | 1 | 0 | 65.64 |
| 14 | 0 | 0 | 0 | 77.83 |
| 15 | 1 | -1 | 0 | 65.44 |
| 16 | 0 | 1 | 1 | 70.11 |
| 17 | 0 | 0 | 0 | 77.41 |

Table S3 Analysis of variance and significance test of regression model

| Source | Sum of Squares | DF | Mean Square | F-value | p-value | Significant |
| --- | --- | --- | --- | --- | --- | --- |
| Model | 344.712125 | 9 | 38.301347222222 | 39.475024283508 | 3.5428752024868e-05 | ** |
| A-Time | 30.3031125 | 1 | 30.3031125 | 31.231697800681 | 0.00082580751604253 | ** |
| B-Ultrasonic power | 11.4481125 | 1 | 11.4481125 | 11.798919665041 | 0.01091158913975 | * |
| C-Enzyme addition amount | 0.0018000000000029 | 1 | 0.0018000000000029 | 0.0018551578172479 | 0.96684736274501 | - |
| AB | 5.8563999999999 | 1 | 5.8563999999999 | 6.0358590227292 | 0.043669784889272 | * |
| AC | 0.29702499999996 | 1 | 0.29702499999996 | 0.30612680592616 | 0.59728605387884 | - |
| BC | 0.455625 | 1 | 0.455625 | 0.4695868224901 | 0.51522125348881 | - |
| A² | 150.44423684211 | 1 | 150.44423684211 | 155.05433446504 | 4.9589966554075e-06 | ** |
| B² | 77.581289473684 | 1 | 77.581289473684 | 79.958630910579 | 4.4489530365026e-05 | ** |
| C² | 39.813157894737 | 1 | 39.813157894737 | 41.033161720903 | 0.00036529159354304 | ** |
| Residual | 6.791875 | 7 | 0.97026785714286 |  |  |  |
| Lack of Fit | 4.735075 | 3 | 1.5783583333333 | 3.0695416828731 | 0.15346594493653 | - |
| Pure Error | 2.0568 | 4 | 0.5142 |  |  |  |
| Cor Total | 351.504 | 16 |  |  |  |  |
| R^2^ | 0.9807 |  |  |  |  |  |
| R^2^Adj | 0.9558 |  |  |  |  |  |
| R^2^Pred | 0.7753 |  |  |  |  |  |
| C.V.% | 1.39 |  |  |  |  |  |
| S/N | 16.19 |  |  |  |  |  |

Table S4 Results of score data of different loach peptides

| No. | Peptide | Activity score | Hydrophobicity score | Toxicity | Sensitizing potential | Free radical scavenger (FRS) | Chelation score |
| --- | --- | --- | --- | --- | --- | --- | --- |
| 1 | GFGGGMGGGFGGGMG | 0.95374 | 25.96 | Non-Toxin | Non-Allergen | 0.53663498 | 0.18531224 |
| 2 | FGGGMGGGFGGGMG | 0.94607 | 24.71 | Non-Toxin | Non-Allergen | 0.53571606 | 0.18581381 |
| 3 | SGFGGGGGYGFGGGSGFGG | 0.92685 | 28.29 | Non-Toxin | Non-Allergen | 0.5939188 | 0.16708724 |
| 4 | SGFGGGGYGFGGGSGF | 0.92679 | 29.15 | Non-Toxin | Non-Allergen | 0.65016526 | 0.15910529 |
| 5 | GGYGGFGGMGGGFGGMGGGMG | 0.86424 | 32.28 | Non-Toxin | Non-Allergen | 0.52811193 | 0.17692253 |
| 6 | FMWNEHLGF | 0.88226 | 37.56 | Non-Toxin | Non-Allergen | 0.57208538 | 0.22816628 |
| 7 | SGFGGGGGYGFGGGSG | 0.83791 | 21.2 | Non-Toxin | Non-Allergen | 0.6363253 | 0.15864259 |
| 8 | AGGMPGGMPDGMPGGFPGAG | 0.82952 | 29.89 | Non-Toxin | Probable Allergen | 0.53799075 | 0.22568542 |
| 9 | EEKEMPSGGMGGMGGMGGMG | 0.81652 | 26.14 | Non-Toxin | Non-Allergen | 0.51031005 | 0.16525008 |
| 10 | GSGGGGAGGGGASGGGGS | 0.79842 | 0.02 | Non-Toxin | Non-Allergen | 0.442177 | 0.18175295 |
| 11 | MPPVPPLPGQP | 0.78093 | 24.44 | Non-Toxin | Non-Allergen | 0.58161485 | 0.30844197 |
| 12 | LYPPSADFPDLR | 0.76033 | 30.35 | Non-Toxin | Non-Allergen | 0.41840214 | 0.21380691 |
| 13 | LHVDPDNFRL | 0.74771 | 27.55 | Non-Toxin | Probable Allergen | 0.40220469 | 0.27501443 |
| 14 | FDAVIFPGGHG | 0.73672 | 27.60 | Non-Toxin | Probable Allergen | 0.43087363 | 0.24137217 |
| 15 | IADHFLFDKP | 0.73507 | 30.34 | Non-Toxin | Probable Allergen | 0.39310369 | 0.27862364 |
| 16 | EEKEMPSGGMGGMGGMGG | 0.73041 | 21.66 | Non-Toxin | Non-Allergen | 0.48809236 | 0.17080361 |
| 17 | LDPVYPPGPPAFPK | 0.72949 | 30.65 | Non-Toxin | Non-Allergen | 0.64237309 | 0.28744438 |
| 18 | FEVDTFKPF | 0.72547 | 33.06 | Non-Toxin | Probable Allergen | 0.39585808 | 0.23122306 |
| 19 | LLPKAPGGEEPLPEGL | 0.71256 | 32.23 | Non-Toxin | Non-Allergen | 0.48776695 | 0.21503063 |
| 20 | DIGAEALGRML | 0.71147 | 29.81 | Non-Toxin | Non-Allergen | 0.33259809 | 0.23064047 |

Table S5 The binding ability between the peptide sequence and the Keap1-Nrf2 receptor

| ID | Peptide | Binding energy(KJ/mol) |
| --- | --- | --- |
| 1 | GG-15 | -55.82 |
| 2 | FG-14 | -86.43 |
| 3 | SG-19 | -70.23 |
| 4 | GG-16 | -25.1 |
| 5 | GG-21 | -44.18 |
| 6 | FF-9 | -42.93 |
| 7 | SG-16 | -30.02 |
| 8 | AG-20 | -67.15 |
